# Efficacy of publicly accessible tourniquets: a systematic review of layperson performance utilizing simulation models

**DOI:** 10.1186/s41077-025-00390-y

**Published:** 2025-11-18

**Authors:** Steven Bordonaro, Christopher Negro, Karl Neubecker, Eric C. Nemec, Suzanne J. Rose

**Affiliations:** 1 Master of Physician Assistant Studies, Sacred Heart University, 5151 Park Ave, Fairfield, CT 06825 USA; 2 Orthopedic Associates of Hartford, 31 Seymour St #100, Hartford, CT 06106 USA; 3 Department of Research and Discovery, Stamford Health, One Hospital Plaza, Stamford, CT 06902 USA

**Keywords:** Tourniquet, Laypeople, Hemorrhage control, Stop the Bleed, Point-of-care, Effective application, Manikin

## Abstract

**Background:**

A large portion of preventable deaths is a result of uncontrolled bleeding due to a delay in medical intervention. While publicly accessible tourniquets raise the concern of incorrect application by laypeople, tourniquets have proven efficacy and can be effectively applied by bystanders. This systematic review aims to identify if tourniquets applied by laypeople using a basic manikin or tourniquet trainer extremity with little to no training can effectively control bleeding.

**Methods:**

The authors used EBSCOHost to simultaneously search the following databases: Cumulated Index in Nursing and Allied Health Literature (CINAHL) Ultimate, Academic Search Premier, Cochrane Central Register of Controlled Trials, Cochrane Database of Systematic Reviews, and Medical Literature Analysis and Retrieval System Online (MEDLINE) with Full Text. Boolean search strategy included tourniquet AND (layperson OR laypeople) AND ((bleeding AND control) OR (hemorrhage AND control) OR "stop the bleed") NOT surgery. The search was limited to January 1, 2013, to August 31, 2023. Inclusion criteria were layperson participants in peer-reviewed randomized controlled or clinical trials, available in English, that assessed at least one outcome measure related to the efficacy of tourniquet application in a simulated context. Articles including duplicate data and those regarding tourniquet use/efficacy in settings other than prehospital care or bleeding control were excluded. Two independent reviewers selected studies according to prespecified inclusion and exclusion criteria. Risk of bias was assessed using the Cochrane RoB 2 tool.

**Results:**

The initial search identified 83 studies, with 10 retained for inclusion in this review. Two different windlass rod tourniquets and one ratcheting strap tourniquet performed the best in terms of successful application by laypeople. Completing formal bleeding control training increased the average application success rate compared to no prior training. The Layperson Audiovisual Assist Tourniquet was the only audiovisual point-of-care aid that significantly increased the rate of successful applications. Just-in-Time visual cards also increased success rates significantly, showing comparable benefits to manufacturer instructions.

**Conclusion:**

Although some laypeople can successfully place tourniquets without prior training, successful placement rates can be improved with point-of-care aids and formal bleeding control training using a basic manikin or tourniquet trainer extremity.

**Supplementary Information:**

The online version contains supplementary material available at 10.1186/s41077-025-00390-y.

## Background

Uncontrolled hemorrhage is a leading cause of preventable death in trauma cases, particularly in prehospital settings where immediate medical intervention may be delayed. Studies have shown that a significant number of trauma-related deaths occur before hospital arrival, with hemorrhage being a primary cause [1]. In situations such as mass casualty incidents (MCI), motor vehicle accidents, industrial injuries, or violent attacks, severe bleeding can become fatal within minutes if not addressed promptly. While emergency medical personnel are trained to manage such conditions, the time it takes for them to arrive on the scene can be critical. Therefore, empowering laypeople who do not have professional training, qualifications, or experience in a healthcare-related discipline with the knowledge and skills to apply tourniquets in emergencies is essential for improving survival rates.

The importance of immediate hemorrhage control by bystanders has gained increasing attention in recent years. A strategy for addressing this is the implementation of hemorrhage control kits in public spaces. Stop the Bleed, a program led by the American College of Surgeons, advocates for the distribution of bleeding control supplies and layperson training in how to use those supplies. Kits sold by Stop the Bleed typically include a tourniquet, hemostatic gauze, and a pressure dressing, amongst other items [2]. Though these kits have lifesaving potential, California is the only state that requires accessible bleeding control kits in public and private buildings [3]. In the absence of easily accessible bleeding control kits, holding direct pressure may be the best – and only – option for bystanders when exposed to a patient with an uncontrollable hemorrhage. Despite growing awareness, many individuals remain hesitant to use tourniquets due to misconceptions about their application and potential complications. However, research indicates that when applied correctly, tourniquets are highly effective in controlling extremity hemorrhage, and the risk of complications is minimal compared to the risk of exsanguination [4].

Tourniquets have long been used in the military and are routinely used in prehospital settings. The Combat Application Tourniquet (CAT) specifically is the industry standard for the military and prehospital settings. It is utilized in Stop the Bleed training and is the most popular tourniquet type worldwide [5]. While publicly accessible tourniquets raise the concern of incorrect application by laypeople, tourniquets have proven efficacy when applied by trained professionals. In Iraq and Afghanistan operations alone, tourniquets prevented mortality in an estimated 1000 to 2000 members of the United States (US) military [6].

While mass casualty events garner attention, everyday trauma events remain the most frequent scenarios necessitating tourniquet application. Prehospital use of tourniquets for hemorrhage control in the civilian sector is associated with improved survival and fewer blood transfusions [7]. Applying tourniquets prehospitally as opposed to applying them upon arrival at a trauma center has been associated with a 4.5-fold decrease in mortality [8]. Assuming tourniquets are more prone to user error when applied by a layperson, these statistics at least represent a *potential* benefit of easily accessible tourniquets, given that a layperson has the *potential* to apply the tourniquet just as successfully as a trained professional. Regarding the safety of tourniquet use, the chance of tourniquet-related morbidity and the possible adverse effects are both deemed minor, especially compared to the potential survival benefits of applying a tourniquet [7, 9].

Previous systematic reviews have focused on important aspects of hemorrhage control using the Stop the Bleed program. Two recent reviews have evaluated the effectiveness of the Stop The Bleed training course for laypeople concluding that it significantly increases participants' knowledge of hemorrhage control and their ability to apply skills like using a tourniquet, empowering them with the knowledge and confidence to control life-threatening bleeding [10, 11]. In addition, a systematic review by Nichols and Horstman (2022) focuses on the impact of the Stop the Bleed program like the aforementioned publications, with subsequent recommendations on how it can be improved [12].

While the aforementioned systematic reviews offer valuable research to the field, the current body of literature, to the best of our knowledge, is lacking in systematic reviews focused on evaluating the efficacy of publicly accessible tourniquets, with formal bleeding control training being only one of the variables considered. The measures of knowledge gained and confidence/willingness of a layperson to administer bleeding control, which are outcomes emphasized in the existing literature, does not necessarily translate to the efficacy of said bleeding control. This systematic review therefore aims to identify if tourniquets applied by laypeople with little to no training effectively control bleeding using a basic manikin or tourniquet trainer extremity.

## Methods

### Ethics approval

An ethics statement is not applicable because this study is based exclusively on published literature. The protocol for this systematic review was registered on INPLASY (INPLASY202590097) and is available in full on inplasy.com (10.37766/inplasy2025.9.0097).

### Search strategy

The authors used EBSCOHost to simultaneously search the following databases: Cumulated Index in Nursing and Allied Health Literature (CINAHL) Ultimate, Academic Search Premier, Cochrane Central Register of Controlled Trials, Cochrane Database of Systematic Reviews, and Medical Literature Analysis and Retrieval System Online (MEDLINE) with Full Text using the following Boolean search string: tourniquet AND (layperson OR laypeople) AND ((bleeding AND control) OR (hemorrhage AND control) OR "stop the bleed") NOT surgery. PubMed, Trip, Google Scholar, ClinicalTrials.gov, medRxiv, and various tourniquet websites, including CAT, Rapid Application Tourniquet (RAT), Stretch Wrap and Tuck Tourniquets (SWAT-T), and Special Operations Force Tactical Tourniquets (SOFT-T) were searched separately. The search was limited to January 1, 2013, to August 31, 2023, to capture the modern era of formal layperson tourniquet training programs and the period after major public health campaigns like Stop the Bleed began to gain traction. Layperson or laypeople are those who do not have professional training, qualifications, or experience in a healthcare-related discipline. The authors followed the definitions provided in studies included in this review.

### Study selection

Two reviewers (S.B. and C.N.) used Covidence [13] to independently screen titles and abstracts, perform full-text reviews, and reconcile discrepancies between reviewers for data extraction. If consensus could not be reached, a third reviewer (S.R.) was utilized to reconcile any final discrepancies. Inclusion criteria were layperson participants in peer-reviewed randomized controlled or clinical trials, available in English, that assessed at least one outcome measure related to the efficacy of tourniquet application in a simulated context. Articles including duplicate data and those regarding tourniquet use/efficacy in settings other than prehospital care or bleeding control (e.g., surgical setting) were excluded. Participants who had declared formal professional medical training were excluded from the analysis. The reference lists of included studies were manually searched to identify additional qualifying studies.

### Quality assessment

Included studies were again independently assessed for risk of bias using the Cochrane Risk of Bias (RoB) 2 tool for randomized controlled trials. One study used a crossover study design, and the Cochrane RoB 2 tool specifically for crossover trials was used [14].

## Results

### Study selection

**Fig. 1:**
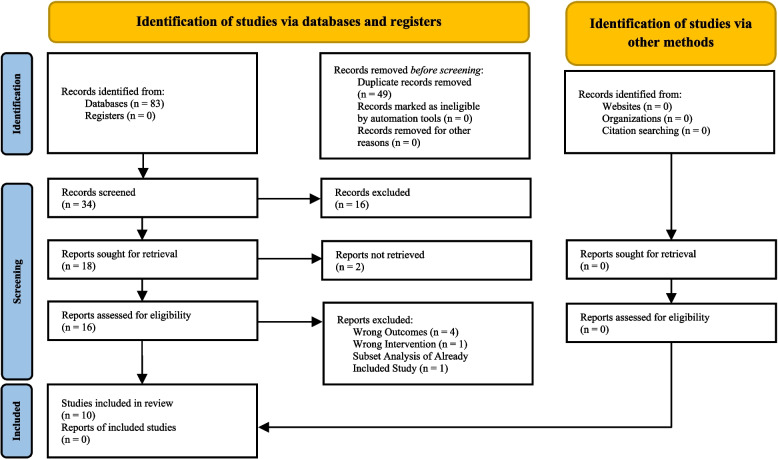
Preferred Reporting Items for Systematic Reviews and Meta-Analyses (PRISMA) Flow Diagram of the studies utilizing simulation that report on the efficacy of publicly accessible tourniquets when employed by laypeople

Following the database search, 83 studies were imported into Covidence. Of the 83 studies identified, 49 duplicates were removed (43 automatically removed by Covidence and 6 removed manually). Two independent reviewers screened the remaining 34 studies by title and abstract, excluding 16 based on the predetermined inclusion and exclusion criteria. Of the 18 remaining studies, the data for 2 studies were inaccessible. The independent reviewers performed full-text reviews on the remaining 16 studies. Authors excluded 6 based on incorrect outcomes, incorrect intervention, and one study that included duplicate data from an already included study. Consensus was reached on 10 studies that were included for analysis [15–24]. The study selection process is depicted below in a Preferred Reporting Items for Systematic Reviews and Meta-Analyses (PRISMA) Flow Diagram (Fig. 1) [25].

### Study characteristics

All studies included in this systematic review analyzed populations in the United Sates of America [15–24], with one study assessing an additional portion of the population in Linköping, Sweden [15]. Studies were published between June 2015 and July 2023. Due to the nature of the studies included through the screening process, it is noted that all studies included the use of a basic manikin or tourniquet trainer extremity. The total population included in the review consists of 1,869 individual laypeople [15–24]. Sixteen individuals were excluded from Dennis et al. (2019) due to prior tourniquet use and/or training [16]. McCarty et al. reported that a portion of the study cohort had prior tourniquet training; however, all participants completed bleeding control (B-Con) training prior to testing; the researchers determined this would have minimal to no effect on the outcome [17].

Materials varied between studies, with the common primary outcome being successful or effective tourniquet application using a basic manikin or tourniquet trainer extremity. All studies defined successful/effective application similarly, including some variation of correct placement and adequate tightness or occlusion of simulated blood flow. Although Dennis et al. reported “patient status” as their primary outcome, they specify that the applied tourniquet would need correct placement and adequate tightness for the manikin leg to read the patient status as “stable” [16]. Tourniquets across all studies were applied to either a basic manikin or tourniquet trainer extremity. The studies using a tourniquet trainer extremity utilized measurements such as time, patient status, tourniquet pressure (mmHg), and estimated blood loss to assess successful tourniquet application [16–18]. The remaining studies using manikins evaluated measures such as time, indentation, and ability to insert fingers to assess tightness and provide a ruling on successful tourniquet application [15, 19–24]. The studies were organized (see Table 1) into three groups based on study characteristics to evaluate trends between tourniquet types [15–24], training interventions[15–24], and benefits of Point-of-Care (POC) aids [15, 16, 19–24].

**Table 1:** Summary of the studies utilizing simulation that report on the efficacy of publicly accessible tourniquets when employed by laypeople [15–24].

Author and Year	Study Design	Study Population (N)	Intervention	Comparisons	Primary Outcomes
Dennis et al. 2019 [16]	Prospective randomized study	150 laypeople (16 of which had prior training/use of tourniquets s	SAM XT, CAT, and SOFT-T. Given manufacturer instructions	Inter-experimental comparisons between tourniquets	Patient status
Gabbitas et al. 2023 [18]	Prospective randomized observational study	84 laypeople	CAT and STAT. Provided standardized manufacturer instructional videos	Inter-experimental comparisons between tourniquets	Successful tourniquet application
Goolsby et al. 2015 [19]	Prospective randomized study	194 laypeople	Just-in-Time instruction card and no instruction with CAT	No Just-in-Time instruction card	Successful tourniquet application
Goolsby et al. 2022 [15]	Prospective randomized superiority study	146 laypeople	LAVA tourniquet with instruction card and CAT with manufacturer's instructions	CAT with manufacturer's instruction card	Successful tourniquet application
Goolsby et al. 2018 [20]	Prospective randomized study	226 laypeople	Preexposure education and no preexposure education. Portion also received looping audio instructions. Given Just-in-Time card and placed a Just-in-Time colored CAT	No preexposure education	Successful tourniquet application
Goolsby et al. 2016 [21]	Prospective randomized study	157 laypeople	Colored CAT and black CAT. Provided Just-in-Time card	Black CAT with Just-in-Time card	Successful tourniquet application
Goralnick et al. 2018 [22]	Prospective randomized clinical trial	465 laypeople	Audio kits with visual aids on tourniquet, instructional flashcards, B-Con training, and no training or instruction with CAT	No training or point-of-care application aids	Successful tourniquet application
McCarty et al. 2019 [17]	Nonblinded, crossover, sequential, intention-to-treat randomized clinical trial	102 laypeople	Different tourniquet s: CAT, SOFT-T, SWAT-T, RATS, and improvised tourniquet after B-Con training	CAT application	Successful tourniquet application
Portela et al. 2020 [23]	Prospective randomized study	166 laypeople	CAT, SOFT-T, SWAT-T, and RMT. Given respective manufacturer instructions	Inter-experimental comparisons between tourniquets	Effectiveness of tourniquet application
Ross et al. 2018 [24]	Prospective randomized study	195 laypeople	12 study arms including tourniquet (CAT, SWAT-T, and RMT), adult or child manikin, and upper or lower extremity	Inter-experimental comparisons between variables	Successful tourniquet application

Abbreviations: *SAM XT* SAM Extremity Tourniquet, *CAT* Combat Application Tourniquet, *SOFT-T* Special Operations Force Tactical Tourniquets, *STAT* Smart Tactical Application Tourniquet, *LAVA* Layperson Audiovisual Assist, *B-Con* Bleeding Control, *SWAT-T* Stretch Wrap and Tuck Tourniquets, *RATS* Rapid Application Tourniquet System, *RMT* Ratcheting Medical Tourniquet

### RoB 2 cochrane risk of bias assessment

Nine studies were assessed using the standard RoB 2 Cochrane tool for randomized trials (see Table 2) [15, 16, 18–24], and one [17] was assessed using the modified RoB 2 tool [14] specifically for randomized crossover trials (see Table 3). Three studies were rated to have some concerns of bias [15, 20, 21] and three studies were rated to have a high risk of bias [17, 23, 24]. Both risk-of-bias tables were created using the robvis tool [26].

**Table 2:**
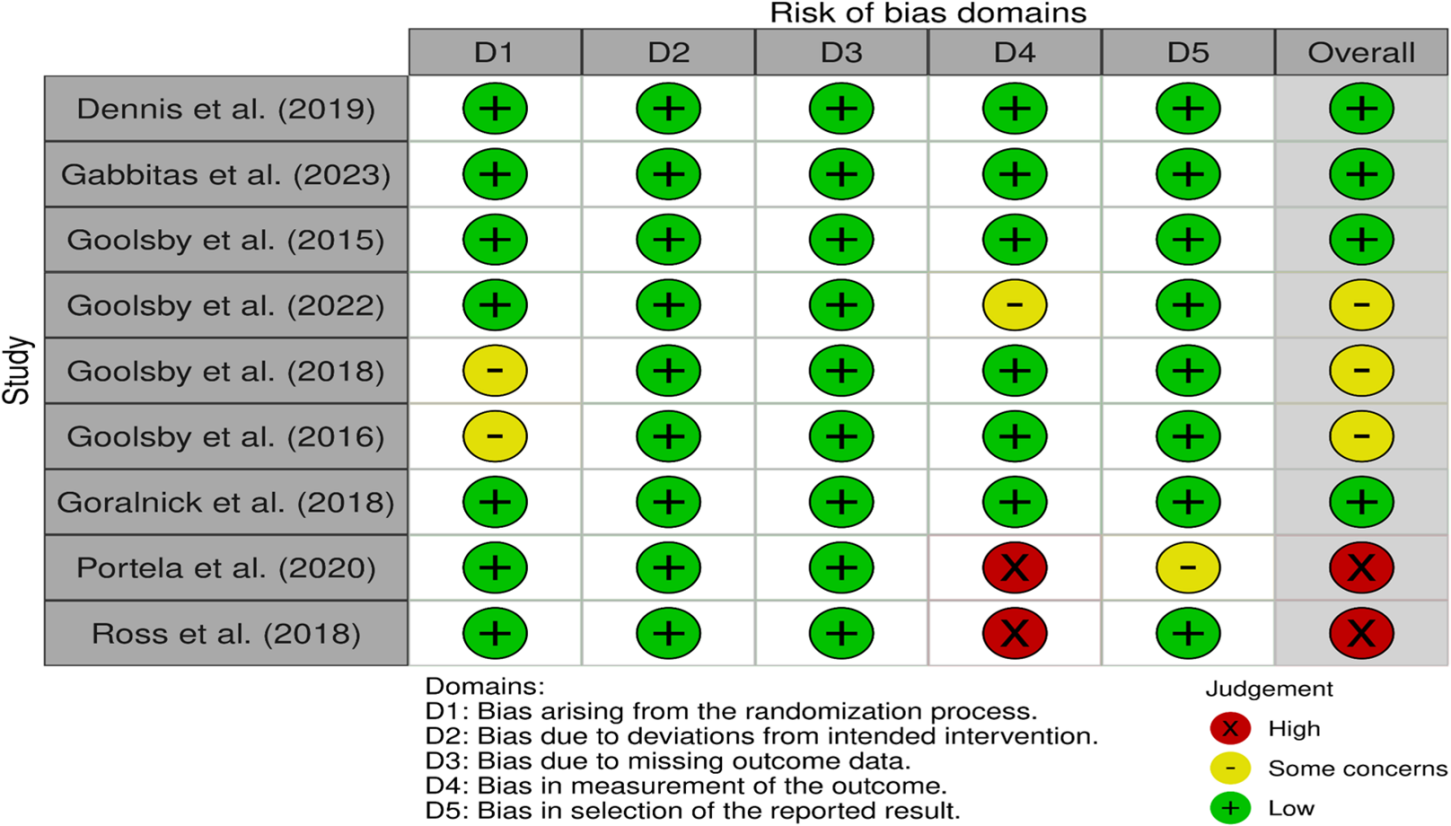
Risk of Bias (RoB) 2 Cochrane Quality Assessment Scale for Randomized Trials of the studies utilizing simulation that report on the efficacy of publicly accessible tourniquets when employed by laypeople [15–24].

**Table 3:**
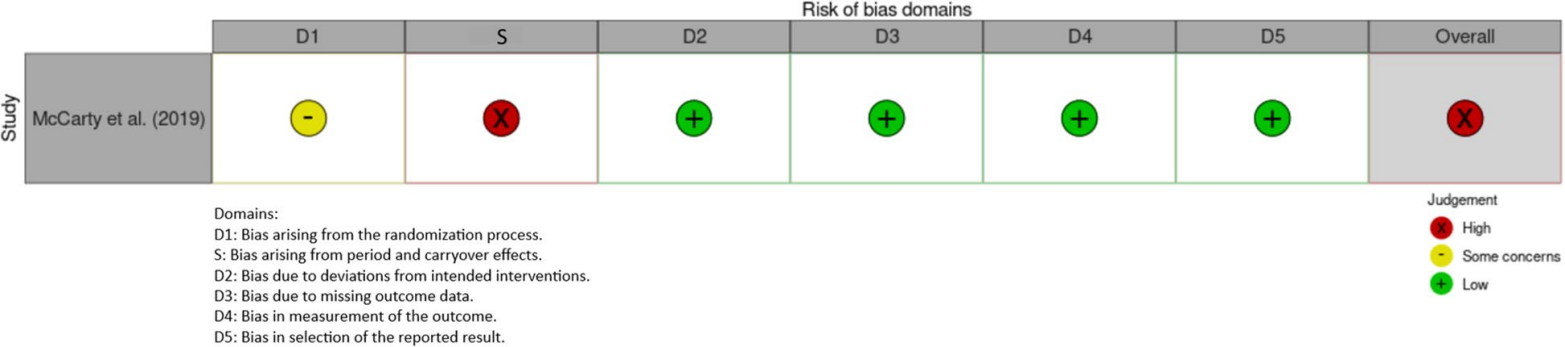
Risk of Bias** (**RoB) 2 Cochrane Quality Assessment Scale for Randomized Crossover Trials of the studies utilizing simulation that report on the efficacy of publicly accessible tourniquets when employed by laypeople [17].

### Efficacy of tourniquets applied by laypeople: effect of tourniquet type

**Fig. 2:**
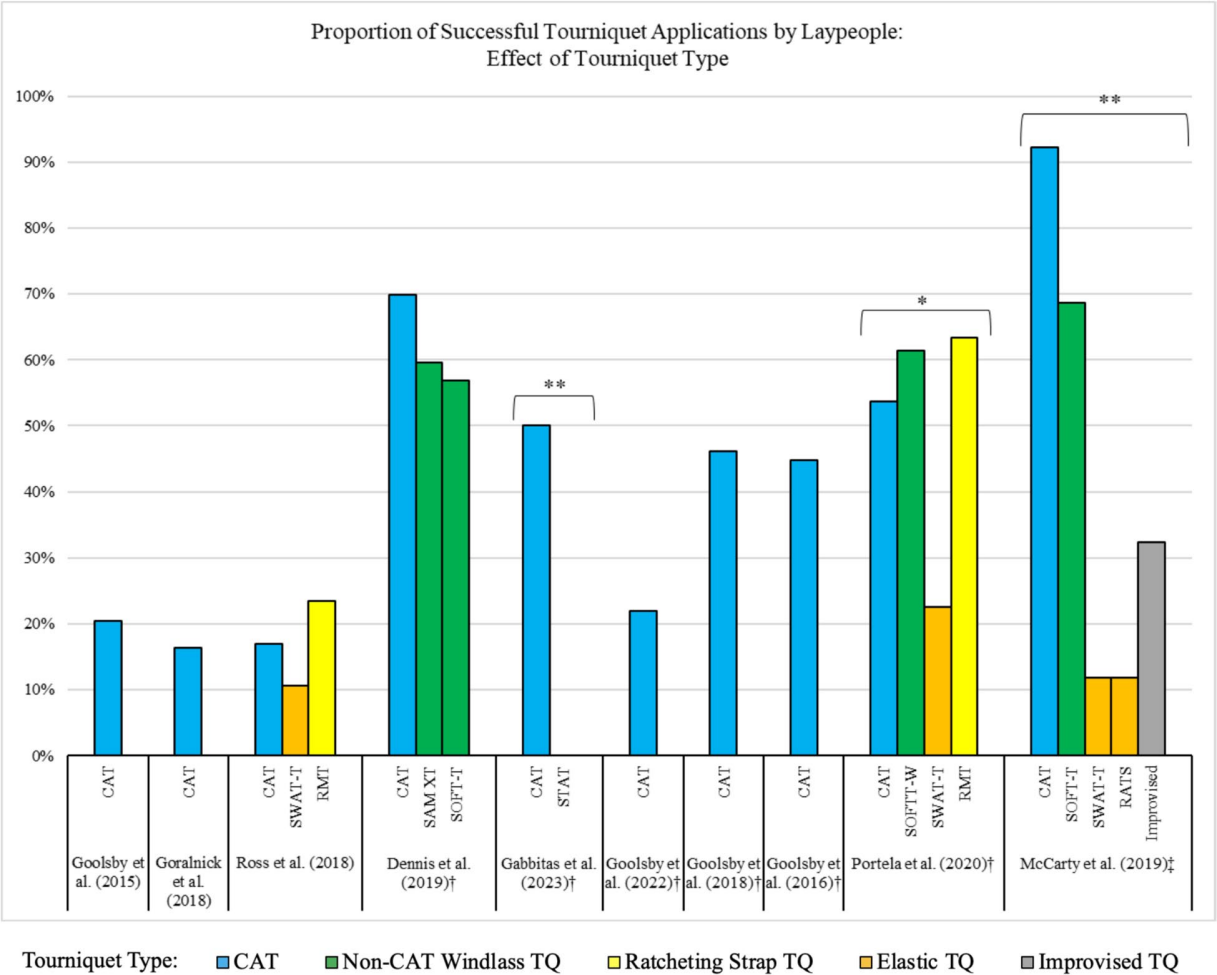
Efficacy of different commercially available and improvised tourniquets [15–24]. Abbreviations: CAT: Combat Application Tourniquet; SWAT-T: Stretch Wrap and Tuck Tourniquets; RMT: Ratcheting Medical Tourniquet; SAM XT: SAM Extremity Tourniquet; SOFT-T: Special Operations Force Tactical Tourniquet; SOFTT-W: Special Operations Force Tactical Tourniquet (Wide); STAT: Smart Tactical Application Tourniquet; RATS: Rapid Application Tourniquet System; TQ: Tourniquet. †Studies where all participants were provided with a point-of-care aid during tourniquet application. ‡Studies where all participants underwent bleeding control training before tourniquet application. *Results with statistical significance (*p* < 0.05). **Results with statistical significance (*p* < 0.001)

Across the ten studies, eight different tourniquet types were assessed, seven of which are commercially available tourniquet types, with the eighth being an improvised tourniquet [15–24]. Figure 2 depicts the efficacy of each tourniquet type, with the tourniquets color-coded by their mechanism: CAT, Non-CAT windlass rod (SAM Extremity Tourniquet (SAM XT) and SOFT-T), ratcheting strap (Ratcheting Medical Tourniquet (RMT) and Smart Tactical Application Tourniquet (STAT)), and elastic (SWAT-T and RATS). Three studies assessed tourniquet efficacy without providing participants with any bleeding control training/instruction beforehand or any point-of-care aids to assist during application [19, 22, 24]. In six of the studies, all participants were provided with some form of point-of-care aid during tourniquet application [15, 16, 18, 20, 21, 23]. McCarty et al. required all participants to undergo bleeding control training before tourniquet application [17]. As shown in Fig. 2, of the five studies that compared different tourniquet types [16–18, 23, 24], three had statistically significant variances in efficacy between tourniquet types, with the CAT being the most successful [17, 18, 23].

For the data shown in Fig. 2, tourniquet type efficacy can be assessed across multiple studies. Across the three studies with no bleeding control training or point-of-care aids, all tourniquet types tested had comparable low success rates ranging from 10.6% to 23.4% [19, 22, 24]. Across the six studies where participants were provided with a POC aid, all windlass-type and ratcheting strap tourniquets had higher success rates [16, 20, 21, 23], with the outliers being the STAT tested in Gabbitas et al. [18] and the CAT tested in Goolsby et al. (2022) [15]. Across all studies where elastic-type tourniquets were tested, they performed worse than the comparator tourniquet types [17, 23, 24].

### Efficacy of tourniquets applied by laypeople: effect of training

**Fig. 3:**
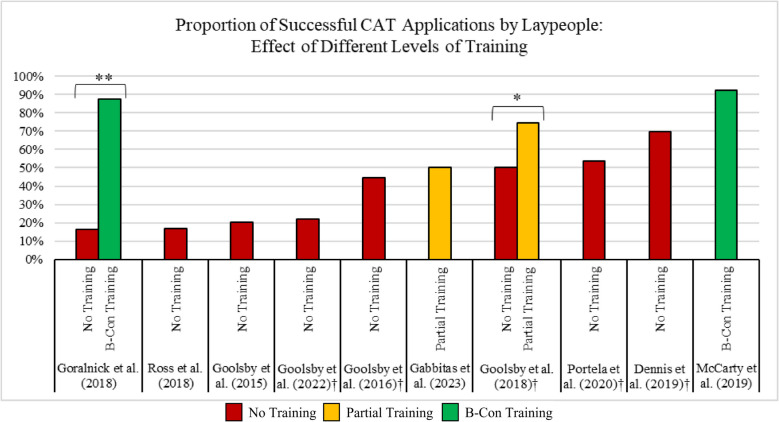
CAT tourniquet specific data from all studies to evaluate application success rates with various levels of training being the independent variable [15–24]. Abbreviations: B-Con: Bleeding Control. †Studies where all participants were provided with a point-of-care aid during tourniquet application. *Results with statistical significance (*p* < 0.05). **Results with statistical significance (*p* < 0.001)

Next, we sought to delineate the success of CAT application stratified by level of training (see Fig. 3 [15–24]). Two studies involved inter-experimental comparisons between levels of training [20, 22]. Three studies contained groups not receiving any training before the evaluation of tourniquet application, ranging in success rates from 16.3% to 20.4% [19, 22, 24]. Five studies included arms with interventions without training but provided participants with POC tools and materials such as instruction cards and audio aids. The success rate of correct tourniquet application in these studies ranged from 21.92% to 69.8% [15, 16, 20, 21, 23]. Partial training with manufacturer instructional videos and website-based education was used in two studies with success rates of 50.00% and 74.70%, respectively. [18, 20]. The last two studies included formal B-Con training, in which participants achieved application success rates of 87.7% and 92.2% [17, 22].

The 3 lowest correct tourniquet application success rates were identified in participants with no training and ranged from 16.3% to 20.4% [19, 22, 24]. The next 7 data points in Fig. 3, with increasingly greater success rates, contain interventions that involved no training but utilized POC materials and interventions categorized as partial training with variable success rates ranging from 21.9% to 74.7% [16, 18, 20, 21, 23]. Finally, the highest rates of successful application consisted of studies with formal B-Con training; 87.7% and 92.2% [17, 22].

### Efficacy of tourniquets applied by laypeople: effect of point-of-care interventions

**Fig. 4:**
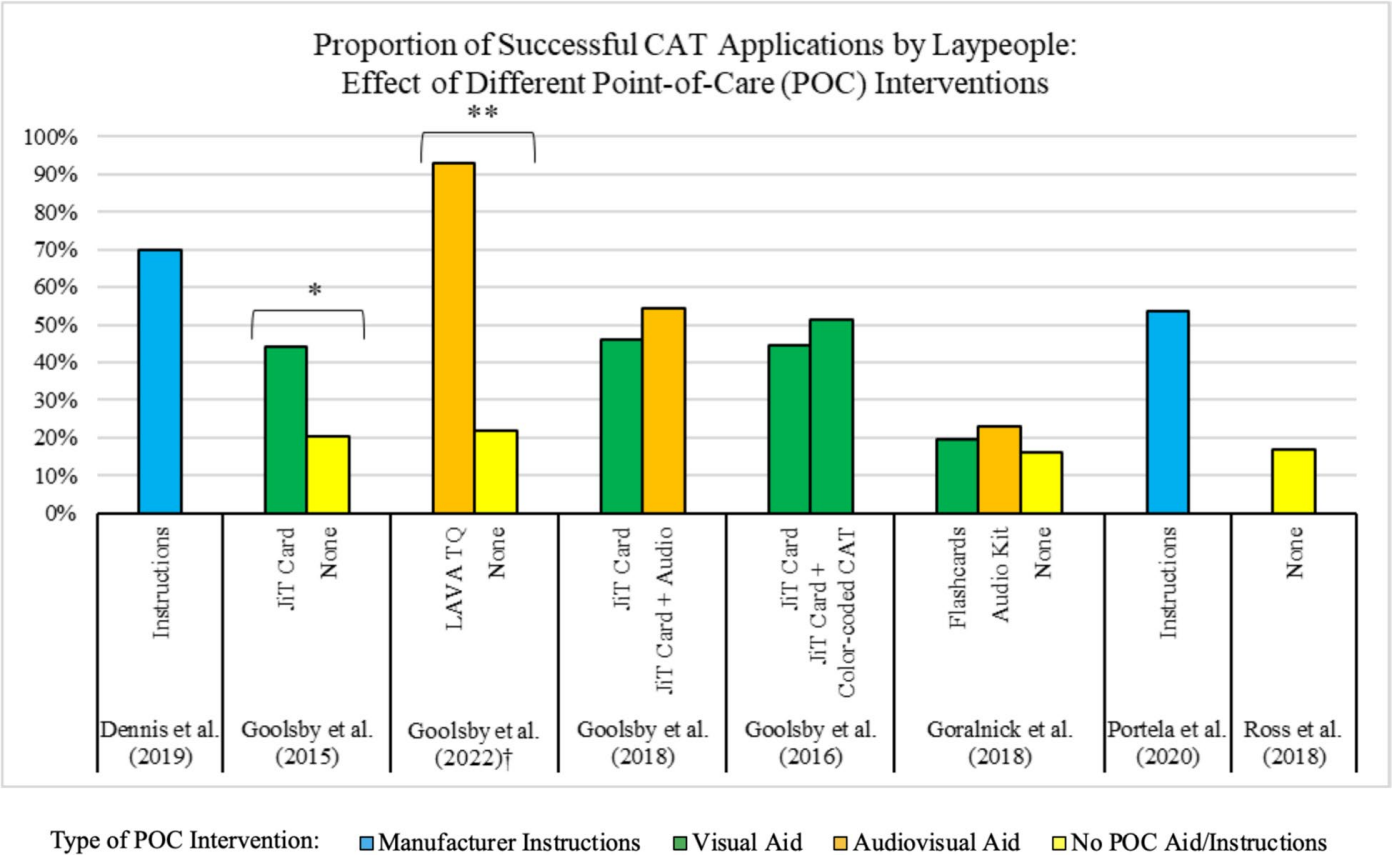
Efficacy of CAT application with different point-of-care interventions [15, 16, 19–24]. Abbreviations: JiT: Just-in-Time LAVA TQ: Layperson Audiovisual Assist Tourniquet; CAT: Combat Application Tourniquet; POC: Point-of-Care^.^ †A tourniquet type other than the CAT used by experimental group; CAT used by control group. *Results with statistical significance (*p* < 0.05).^.^ **Results with statistical significance (*p* < 0.001)

Eight of the ten studies assessed the effect of POC interventions (or lack thereof) on the efficacy of tourniquet application (Fig. 4) [15, 16, 19–24]. The POC interventions included manufacturer instructions, visual aids, and audio aids. Five of these studies directly compared POC interventions to one another or to a control, with the CAT being the tourniquet used in all five [15, 19–22]. Of the three studies that compared different POC aids to no aid, two demonstrated statistically significant results, with a POC intervention outperforming no intervention (Fig. 4) [15, 19, 22].

## Discussion

This review shows that laypeople can effectively apply tourniquets, especially when equipped with training or point-of-care materials. The CAT, SOFT-T, and RMT were shown to be the most promising tourniquet type options for laypeople. The windlass tourniquets performed relatively consistently throughout all studies [15–24]. The RMT performed slightly better than the CAT and SOFT-T; however, there are less data compared to the CAT and SOFT-T to support its efficacy. While the RMT showed marginally higher success in a limited sample, widespread implementation of a novel device would require substantial additional data, cost–benefit analyses, and training modifications. Non-CAT windlass tourniquets performed similarly to the CAT [16, 23]: however, the SAM XT or SOFT-T would need to show a significant improvement over the CAT to justify replacing it as the CAT is currently the industry standard and already implemented in Stop the Bleed Training as well as most commercially available bleeding control kits [5]. Until further research is conducted, we recommend efforts to make the CAT publicly accessible continue, as it has the most data supporting its efficacy in the hands of military personnel, pre-hospital providers, and laypeople.

The LAVA tourniquet and Just-in-Time instruction cards were both shown to be promising POC interventions [15, 19]. The LAVA tourniquet, however, is a novel design, so further testing is needed before a product like this becomes widely available. While the Just-in-Time cards would be much easier and more cost-efficient to implement, it is worth noting that where manufacturer instructions were utilized [16, 23], they yielded a better tourniquet application rate than any study where Just-in-Time cards were used [19–21]. Further research is merited to compare supplemental visual aids like the Just-in-Time cards to manufacturer instructions, as none of the included studies made that comparison. Previous systematic reviews have focused mainly on the Stop the Bleed training and the demonstrated increase in participants' knowledge of hemorrhage control techniques [10–12]. While these reviews advocate for larger scale Stop the Bleed training, which is supported by our review as well, our review provides evidence for the potential benefit of accessible tourniquets even for persons lacking Stop the Bleed training. While formal bleeding control training, like Stop the Bleed, may be the gold standard, our review provides evidence that other interventions like visual aids/diagrams can augment tourniquet application success rates to a degree.

In the United States of America, there has been a notable increase in publicly available life-saving devices within the last decade, such as automated external defibrillators (AEDs). In fact, in the United States of America, all 50 states have some form of law that addresses public access defibrillation (PAD), with a majority of states authorizing certain evidence-based interventions to enhance PAD; 37 states specifically require AED placement at locations where out-of-hospital cardiac arrests are most likely to happen, and 19 states require/encourage AED placement to be clearly marked or easily accessible [27]. The authors believe that tourniquets should similarly be publicly accessible. With the substantially lower price point of a bleeding control kit than an AED – individual kits costing around $100 – having these kits co-located with AEDs on a large scale could be achieved relatively easily [28].

### Review limitations

This manuscript acknowledges several limitations, primarily stemming from the high risk of bias present in several of the included studies [17, 23, 24]. These factors may limit the generalizability of the findings and introduce variability in the reported outcomes. Additionally, inconsistencies in study protocols and measurement techniques across different sources make direct comparisons challenging and the study unsuitable to perform a meta-analysis. Different manikin types were used to facilitate training across studies, which may have impacted the tourniquet application success rate. A below-the-knee amputation model like that of Goolsby et al. (2022) [15] could make placement location more intuitive compared to a full-body model like the one used by Ross et al. [24].

Most of the included studies were performed in the United States of America, potentially limiting generalizability. Moreover, none of the studies assessed patient-centered clinical outcomes (e.g., limb ischemia, mortality), as they were simulation-based. In addition, the simulated nature of included studies could not possibly assess how a layperson would perform with the stress and chaos of a true MCI [29]. The authors acknowledge that the lack of stress and chaos in a simulated environment is perhaps the largest barrier for determining the benefit of publicly accessible tourniquets. Three studies reported that the ability to recognize the indications of tourniquet use was not assessed, and doing so was a limitation of their study [19, 21, 22]. Four studies reported the possibility of self-selection bias as a limitation, given that people who are more willing to use a tourniquet or have a baseline knowledge of tourniquet use from the media may be more likely to volunteer for these studies [17, 20, 21, 23].

In addition, the narrow scope of this research is whether publicly accessible tourniquets can be effectively applied by laypeople with varying levels of training and instruction material. We understand that there may be other options for bleeding control, such as direct pressure or wound packing, though tourniquets require more technical expertise compared to the other modalities of bleeding control. Direct pressure applied by a layperson has a clearer benefit given that the technique logically has a smaller margin for user error compared to the deployment of a tourniquet by an untrained person; consequently, the larger gap in knowledge lies in layperson tourniquet use rather than the use of publicly accessible gauze for direct pressure or wound packing. We therefore aimed to direct our focus on whether tourniquets available in public spaces, much like AEDs, may provide a potential benefit to rendering aide even if those utilizing the device did not have formal training.

Another limitation of this review is the potential for bias introduced by including multiple articles authored by the same individual, which may influence the overall findings and interpretations. In addition, the conclusions are based on only 10 studies, limiting the generalizability of the findings. Finally, it is possible that articles were missed, as the search databases utilized with the chosen keywords may not have captured all existing studies. Future research should prioritize well-designed, randomized controlled trials and larger prospective studies to provide more robust and reliable evidence on clinical measures. We recommend these studies utilize currently available advanced patient simulators with realistic bleeding consistent with wounds that respond to applied pressure, packing, and wound dressing, along with limbs that stop bleeding when a tourniquet is properly applied to more closely emulate an actively hemorrhaging patient.

## Conclusion

The results of this systematic review, which specifically sought to identify the effectiveness of publicly accessible tourniquets when applied by laypeople with little to no formal training, underscore the significant potential benefits of these programs. The evidence, derived from studies utilizing basic manikins or dedicated tourniquet trainer extremities for simulation, demonstrates that laypersons can successfully apply these devices and achieve effective hemorrhage control. By enhancing public awareness and training, we can bridge the gap between injury occurrence and professional medical care, ultimately reducing preventable deaths due to hemorrhage.

## Supplementary Information

Supplementary Material 1

## Data Availability

The data that support the findings of this study are available through the published articles cited in this systematic review.

## Abbreviations

B-Con: Bleeding Control
CAT: Combat Application Tourniquet
CINAHL: Cumulated Index in Nursing and Allied Health Literature
EMS: Emergency Medical Services
JiT: Just-in-Time
LAVA: Layperson Audiovisual Assist
MCI: Mass Casualty Incidents
MEDLINE: Medical Literature Analysis and Retrieval System Online
POC: Point-of-Care
PRISMA: Preferred Reporting Items for Systematic Reviews and Meta-Analyses
RATS: Rapid Application Tourniquet System
RMT: Ratcheting Medical Tourniquet
RoB: Risk of bias
SAM XT: SAM Extremity Tourniquet
SOFT-T: Special Operations Force Tactical Tourniquets
STAT: Smart Tactical Application Tourniquet
SWAT-T: Stretch Wrap and Tuck Tourniquets
TQ: Tourniquet
